# The Effect of Discrimination on the Daily Life Satisfaction of People with Developmental Disability: Parallel Multiple Mediating Effect of Social Involvement and Self-Esteem

**DOI:** 10.3390/medicina59111988

**Published:** 2023-11-11

**Authors:** Jinhyeok Choi, Byeolhae Shin, Bum-Sung Choi

**Affiliations:** 1 Department of Special Education, Pusan National University, Busan 46241, Republic of Korea; drjinhyeokchoi@gmail.com (J.C.); shinbyeolhae@gmail.com (B.S.); 2 Department of Psychiatry, Pusan National University Yangsan Hospital, Pusan National University School of Medicine, Medical Research Institute, Yangsan 50612, Republic of Korea

**Keywords:** autism spectrum disorder, discrimination, social involvement, self-esteem, daily life satisfaction

## Abstract

*Background and Objectives*: People with developmental disabilities are exposed to discrimination and it affects their daily life satisfaction. The purpose of this study is to examine the parallel mediating effect of social involvement and self-esteem on the relationship between discrimination and the daily life satisfaction of people with developmental disabilities to improve their daily life satisfaction. *Materials and Methods*: This study used raw data of participants with intellectual disabilities and autism spectrum disorder from a national panel survey of employment for the disabled second-wave fifth survey. First, correlations among variables were identified to determine whether variables are in a relationship, and then PROCESS Macro was conducted to identify the relationship between discrimination and daily life satisfaction and the parallel mediating effect of social involvement and self-esteem. *Results*: Discrimination had a significant negative effect on daily life satisfaction and it was found that social involvement and self-esteem have a significant mediating effect that lowers the effect size of discrimination on daily life satisfaction. Specifically, it was found that self-esteem had a more mediating effect than social involvement. *Conclusions*: To increase the daily life satisfaction of people with developmental disabilities, the potential need to not only decrease discrimination but also increase their social involvement and self-esteem should be considered.

## 1. Introduction

Since the 2000s, South Korea has implemented a policy of deinstitutionalization, which has shifted the lives of people with disabilities from relying on facilities to promoting independent daily lives within their local communities. In line with this change, South Korea enacted the ‘Act on the Prohibition of Discrimination against disabled persons and Remedy against Infringement of Rights’ in 2007 with the purpose of legally prohibiting discrimination against people with disabilities. However, according to a survey [1], even with the enactment of such a law, it is still reported that 55.7% of people with disabilities experience discrimination. Specifically, 52.3% of individuals with intellectual disabilities and 49.9% of those with autism spectrum disorder reported experiencing discrimination, which is higher than what is seen for other disability types.

Several studies have found that experiencing discrimination has a negative impact on daily life satisfaction [2,3,4,5]. According to Lee and Jung [2], developmental disability itself increases experiences of discrimination, which mediates the negative impact of developmental disabilities on daily life satisfaction. Kim and Song [4] found that experiencing discrimination in daily life has a negative impact on the life satisfaction of wage laborers. Lee and Yoo [3] claimed that higher levels of discrimination experienced by individuals with developmental disabilities have been significantly associated with lower levels of daily life satisfaction. Experiences of discrimination among individuals with developmental disabilities can be considered as a factor that significantly affects their daily life satisfaction.

Experiencing discrimination has a negative impact on the daily life satisfaction of individuals with disabilities, and social participation [2,3,6] and self-esteem have been found to have positive effects on daily life satisfaction [7,8,9]. According to Lee and Jung [2], engaging in leisure and social activities has been shown to improve the daily life satisfaction of individuals with developmental disabilities. Among various factors, the level of dependence on others, discrimination, and restrictions on leisure and social activities were found to have the most significant mediating effect on daily life satisfaction. Researchers have argued that when it comes to daily life satisfaction, limitations on leisure and social activities have a greater impact than discrimination itself. Therefore, it is crucial to focus on promoting active participation in society for individuals with developmental disabilities, as this will have a positive influence on their quality of life and satisfaction, rather than solely focusing on addressing discrimination. Social participation is a crucial factor that significantly impacts the daily life satisfaction of individuals with developmental disabilities.

Several studies have found that experiencing discrimination has a negative impact not only on life satisfaction but also on self-esteem [10,11,12] and social participation [3,13]. Previous studies have found a negative relationship between discrimination and self-esteem [14,15]. Lee and Yoo [2] recently reported a negative relationship between discrimination and social participation and also found that social participation moderates the relationship between experiencing discrimination and daily life satisfaction. These findings suggest that the relationship between discrimination and social participation for individuals with developmental disabilities should not be ignored.

People with developmental disabilities face higher levels of discrimination due to their disabilities compared to other types of disabilities. Numerous studies have shown that discrimination has a negative impact on their daily life satisfaction. Despite the negative impact of discrimination on daily life satisfaction, research has shown that factors such as self-esteem and social participation can positively enhance their daily life satisfaction. This study aims to investigate the simultaneous effects of social participation and self-esteem on the relationship between discrimination and the daily life satisfaction of individuals with developmental disabilities. The research questions are as follows:

First, what is the relationship between discrimination, daily life satisfaction, self-esteem, and social involvement?

Second, do social participation and self-esteem have a paralleled mediating effect on the relationship between discrimination and daily life satisfaction?

## 2. Materials and Methods

### 2.1. Participants

This study obtained and analyzed raw data from a panel survey of employment for the disabled 2nd wave 5th survey from the South Korea Employment Agency for Persons with Disabilities. A panel survey of employment for disabled individuals began in 2007, with selected respondents who were registered disabled persons aged 15 to 75, with the purpose of providing basic data on disabled workers, and follow-up studies were conducted annually until 2015. The 2nd wave survey began in 2016 with a newly selected sample, followed by an annual follow-up survey, and then the 5th survey of the 2nd wave was conducted in 2020.

Among the 3995 participants in the sample, this study analyzed responses from individuals with intellectual disabilities and autism spectrum disorder, who were considered to have developmental disabilities under the law ‘Act on Guarantee of Rights of and Support for Persons with Developmental Disabilities’. A total of 383 individuals were selected for analysis.

The study included a total of 383 participants, with 355 (92.7%) having intellectual disabilities and 28 (7.3%) having ASD. More than half of the participants were male (65%) and had graduated from high school (62.7%). The age group with the highest population was 15 to 29, accounting for 41.5% of the total. This was followed by the 30~39 (25.3%) and 40~49 (20.1%) age groups. The majority of participants (86.2%) were single and did not have multiple disabilities (90.6%). The participant characteristics are presented in Table 1.

### 2.2. Design

**Figure 1:**
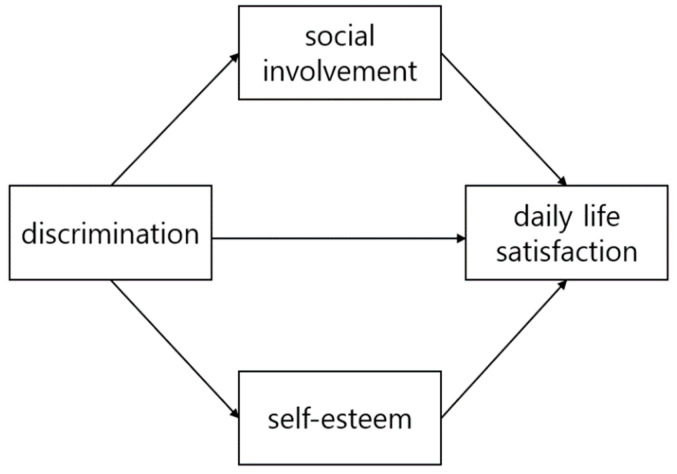
Research model.

This study aims to examine the mediating effects of social involvement and self-esteem on the relationship between daily life discrimination and daily life satisfaction. Therefore, researchers conducted a multiple parallel mediate design to examine the effect size. The research model is presented in Figure 1. 

### 2.3. Variables

For this study, researchers reprocessed the data from ‘The Panel Survey of Employment for the Disabled 2nd wave 5th survey’. Daily life discrimination is an independent variable that directly measures the extent of discrimination experienced in everyday life. There was one question rated on a 4-point Likert scale, ranging from 1 (“did not experience discrimination in daily life”) to 4 (“always experienced discrimination in daily life”). Daily life satisfaction, the dependent variable, was measured on a 5-point Likert scale, ranging from 1 (“very dissatisfied”) to 5 (“very satisfied”). Social involvement, a mediating variable, was assessed on a 4-point Likert scale, ranging from 1 (“not at all”) to 4 (“engaged a lot”). Self-esteem, another mediating variable, consisted of 10 questions about self-evaluation and acceptance. Self-esteem questions were rated on a 4-point Likert scale, ranging from 1 (“not at all”) to 4 (“very much”). Five out of the ten questions were reverse questions which required reverse coding, and the researchers calculated the average of the questions for the study. Except for the self-esteem variable, daily life discrimination, daily life satisfaction and social involvement were assessed using a single direct question to measure the degree of each item.

### 2.4. Data Analysis

The data were analyzed using SPSS version 21 and the PROCESS Macro version 4.2 (Hayes, 2017). The research model used for this study was a mediating model (model 4), which examined the mediating effect of social involvement and self-esteem on the relationship between daily life discrimination and daily life satisfaction. Researchers first examined the correlations among the variables to confirm their relationship and to identify the parallel mediating effects of selected variables. The PROCESS Macro was used to determine the effect size and significance of the paths.

## 3. Results

### 3.1. Correlation

The study examined the correlations among variables to determine the mediating effect of social involvement and self-esteem on the relationship between discrimination and satisfaction with daily life. The study confirmed that discrimination is negatively correlated with social involvement (*r* = −0.12, *p* < 0.05), self-esteem (*r* = −0.17, *p* < 0.01) and satisfaction with daily life (*r* = −0.36, *p* < 0.01). Social involvement was found to have positive correlations with self-esteem (*r* = 0.30, *p* < 0.01) and satisfaction with daily life (*r* = 0.28, *p* < 0.01). The correlation between self-esteem and satisfaction with daily life was found to be positive (*r* = 0.34, *p* < 0.01). The detailed results are shown in Table 2.

### 3.2. Mediating Effect

#### 3.2.1. Direct Effect among Variables

It has been confirmed that discrimination has a negative impact on daily life satisfaction (*B* = −0.211, *p* < 0.001), self-esteem (*B* = −0.081, *p* < 0.01) and social involvement (*B* = −0.107, *p* < 0.05). On the other hand, social involvement (*B* = 0.149, *p* < 0.01) and self-esteem (*B* = 0.089, *p* < 0.001) have a positive effect on daily life satisfaction. Detailed results are shown in Table 3.

#### 3.2.2. Parallel Mediating Effect

**Figure 2:**
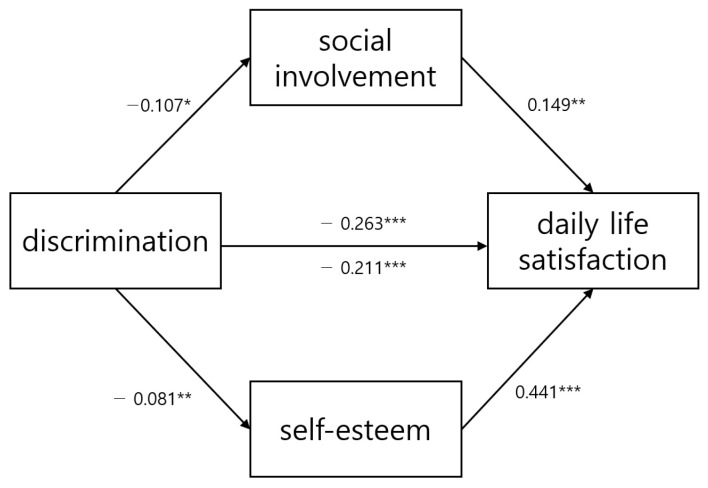
Direct effect and total effect of research model. * *p* < 0.05, ** *p* < 0.01, *** *p* < 0.001.

To assess the statistical significance of the parallel mediating effect of social involvement and self-esteem on the relationship between discrimination and daily life satisfaction, this study conducted 5000 bootstrapping at a *p*-value 0.05 level. As a result, the total effect of the relationship between discrimination and daily life satisfaction was −0.26 [−0.35, −0.18]. When social involvement and self-esteem were included in the analysis, the direct effect of discrimination on daily life satisfaction decreased to −0.21 [−0.29, −0.13]. This finding confirms the statistical significance of the indirect effect of the mediating variables. The total effect (*B* = −0.26, *p* < 0.01) was stronger than the direct effect (*B* = −0.21, *p* < 0.01), suggesting the presence of potential indirect effects in the research model. The detailed total effect and direct effect sizes are presented in Table 4, while a summary of the research model is provided in Figure 2.

#### 3.2.3. Indirect Effects of Mediating Variables

The total indirect effect of social involvement and self-esteem was −0.06 [−0.098, −0.029]. The effect size of social involvement was −02 [−0.042, −0.001] and the effect size of self-esteem was −0.04 [−0.072, −0.018]. No zero values were found between Boot LLCI and Boot ULCI at a 95% level of confidence interval. Therefore, it was found that self-esteem had a greater indirect effect than social involvement (Table 5).

## 4. Discussion

This study investigated the mediating effect of social participation and self-esteem on the relationship between discrimination and daily life satisfaction. The points of discussion based on the results of this study are as follows. First, the results of this study were consistent with previous researches [2,3,4,5] which discrimination had a negative effect on the daily life satisfaction of people with developmental disabilities. Lee and Jeong [2] reported that the developmental disability is a factor that increases the experience of discrimination, and discrimination appears to be a mediating factor in the relationship that affects daily life satisfaction. Therefore, mediating factors that can reduce the negative effects of disabilities should be the key to improving the daily life satisfaction of people with developmental disabilities.

The study results confirmed that both social involvement and self-esteem partially mediated the relationship between discrimination and daily life satisfaction, which eventually reduced the negative effects of discrimination on daily life satisfaction. For the improvement in self-esteem among individuals with developmental disabilities, exercise programs [16,17,18] and leisure activity programs [19] were shown to have effects in South Korea. Horticultural therapy [20] and music therapy [21] were the other effective approaches for improving the self-esteem of people with developmental disabilities in South Korea. Self-esteem is a concept of how one perceives and positively evaluates oneself, and it is one of the possible contributors that makes people happy [22,23]. Therefore, supporting these people to play active roles in their life should be considered to improve their self-esteem. According to Sanders [24] people with disabilities frequently experience overprotection and lowered expectations, which can result in lowered self-esteem. Hence, people with developmental disabilities should have more opportunities to be independent and accomplish their goals.

This study also confirmed that social involvement, as a mediating factor, can lower the negative impact of discrimination on daily life satisfaction. Communication has been identified as a significant factor influencing the social participation of individuals with developmental disabilities [25]. Specifically, individuals with developmental disabilities often encounter communication difficulties, which leads to challenges in accurate communication and social interaction. These limitations in interpersonal skills can restrict their participation in social activities [2,26]. Consequently, it is necessary to provide appropriate individualized communication support for individuals with developmental disabilities.

Furthermore, individuals with developmental disabilities often face challenges in living independently in their daily lives and often require assistance from others. This factor can significantly limit their participation in social activities. According to Ministry of Health and Welfare [27], 75.7% of individuals with developmental disabilities reported needing assistance in their daily lives. Among them, 43.6% required assistance for at least six hours per day. However, only 23.5% of individuals with developmental disabilities received the necessary amount of assistance in their daily lives, with the majority being dependent on their parents (69.7%). In essence, for individuals with developmental disabilities to be more actively involved in the social community, it is crucial to provide support that addresses their daily life needs. Currently, most of the daily life support for individuals with developmental disabilities is provided by their families. Therefore, in order to foster greater participation in social activities for individuals with developmental disabilities, it is essential to establish a comprehensive support system that caters to their specific needs in daily life.

Furthermore, according to Ministry of Health and Welfare [27], the majority of respondents above 15 years old were found to be engaged in activities such as watching TV (21%) and taking rest (17.2%) during weekdays, aside from work (27.3%) and commuting to school (10.4%). Daily activities on the weekend were also found to be similar to weekdays, with a significant portion of people taking a rest (29.3%) and watching TV (28.3%). This suggests that individuals with developmental disabilities engage in a limited range of daily activities. Despite the study’s findings on the impact of social involvement, many individuals with developmental disabilities still spend a significant amount of time at home. Identifying barriers to social engagement and diversifying activities are crucial for increasing social involvement.

The limitations and suggestions of this study are as follows. There was a limitation in creating a balanced sample of respondents within the autism disorder group when reanalyzing the existing panel data. Therefore, future studies should re-evaluate the study’s findings by including a balanced representation of both groups. Additionally, due to the use of panel survey data, some variables could not be measured thoroughly. For example, self-esteem was assessed by averaging the responses to 10 questions, while discrimination, social involvement, and daily life satisfaction were measured using a single question each. Therefore, it is necessary to conduct a thorough analysis of these variables in order to accurately determine their relationships. Third, ‘The Panel Survey of Employment for the Disabled 2nd wave 5th survey’ allowed proxy responses of family members for participants with intellectual disabilities and autism spectrum disorder with consideration of the situation that the participant could not answer the questions. It was found that 17.5% of the participants’ responses were made via proxy response. Therefore, this should be noted carefully when translating the results of the study.

## 5. Conclusions

This study investigated and confirmed the relationship between discrimination and the daily life satisfaction of people with developmental disabilities. The study discovered that self-esteem and social involvement played a mediating role in the effect of discrimination on daily life satisfaction, reducing its negative effects. People with developmental disabilities are more likely to face discrimination compared to other disability groups. The study not only examines the effectiveness of mediators but also compares how these mediators impact the relationship between discrimination and daily life satisfaction. While social involvement requires people with developmental disabilities to participate in the community, what is more important for self-esteem is how they see themselves. In this study, self-esteem had a greater impact as a mediator than social involvement. Therefore, supporting individuals with developmental disabilities in developing healthy self-perception is crucial in reducing the negative impacts of discrimination.

## Figures and Tables

**Table 1:** Characteristics of participants.

Item	Category	*n* (%)	Item	Category	*n* (%)
Disability	ID	355 (92.7)	Academic ability	Uneducated	33 (8.6)
	ASD	28 (7.3)		Elementary	34 (8.9)
	Male	249 (65)		Middle School	51 (13.3)
Sex	Female	134 (35)		High School	240 (62.7)
	Single	330 (86.2)		University	25 (65)
Marriage status	Married/Cohabit	36 (9.4)	Age	15~29	159 (41.5)
	Divorced/	17 (4.4)		30~39	97 (25.3)
	Bereaved			40~49	77 (20.1)
Multiple disabilities	Yes	36 (9.4)		50 and above	50 (13.1)
	No	347 (90.6)			

**Table 2:** Correlations of variables.

	Discrimination	Social Involvement	Self-Esteem	Satisfaction with Daily Life
Discrimination	-			
Social involvement	−0.12 *	-		
Self-esteem	−0.17 **	0.30 **	-	
Satisfaction with daily life	−0.36 **	0.28 **	0.34 **	-

* *p* < 0.05, ** *p* < 0.01.

**Table 3:** Direct effects among variables.

Direct Effect		*B*	SE	*t*
(independent)	(dependent)			
Discrimination → Daily life satisfaction		−0.211	0.041	−5.126 ***
(independent)	(dependent)			
Discrimination → Social involvement		−0.107	0.050	−2.123 *
Discrimination → Self-esteem		−0.081	0.026	−3.133 **
(Mediator)	(dependent)			
Social involvement → Daily life satisfaction		0.149	0.045	3.268 **
Self-esteem → Daily life satisfaction		0.441	0.089	4.970 ***

* *p* < 0.05, ** *p* < 0.01, *** *p* < 0.001.

**Table 4:** Total effect and direct effect of discrimination on daily life satisfaction.

	*B*	SE	*t*	LLCI	ULCI
Total effect	−0.26	0.043	−6.08 ***	−0.35	−0.18
Direct effect	−0.21	0.041	−5.13 ***	−0.29	−0.13
Model summary R = 0.313, R^2^ = 0.098, F = 36.992 ***					

*** *p* < 0.001.

**Table 5:** Indirect effect of discrimination on daily life satisfaction through social activity involvement and self-esteem (Bootstrapping 95% CI).

Category	*B*	Boot SE	95% CI	
			Boot LLCI	Boot ULCI
Discrimination → Social involvement → Daily life satisfaction	−0.02	0.011	−0.042	−0.001
Discrimination → self-esteem → Daily life satisfaction	−0.04	0.014	−0.072	−0.018
Total indirect effect	−0.06	0.018	−0.098	−0.029

## Data Availability

1 March 2023 https://edi.kead.or.kr/ENG_Index.do.
